# The global role, impact, and limitations of Community Health Workers (CHWs) in breast cancer screening: a scoping review and recommendations to promote health equity for all

**DOI:** 10.1080/16549716.2021.1883336

**Published:** 2021-04-26

**Authors:** Taylor Hand, Natalie A. Rosseau, Christina E. Stiles, Tianna Sheih, Elizabeth Ghandakly, Mojisola Oluwasanu, Olufunmilayo I. Olopade

**Affiliations:** a School of Medicine and Health Sciences, The George Washington University, Washington, USA; b Pritzker School of Medicine, University of Chicago, Chicago, IL, USA; c Department of Health Promotion & Education, Faculty of Public Health, University of Ibadan, Ibadan, Nigeria; d Center for Clinical Cancer Genetics & Global Health, The University of Chicago Pritzker School of Medicine, Chicago, IL, USA

**Keywords:** Breast cancer, health policy, global health, community health, health equity

## Abstract

**Introduction**: Innovative interventions are needed to address the growing burden of breast cancer globally, especially among vulnerable patient populations. Given the success of Community Health Workers (CHWs) in addressing communicable diseases and non-communicable diseases, this scoping review will investigate the roles and impacts of CHWs in breast cancer screening programs. This paper also seeks to determine the effectiveness and feasibility of these programs, with particular attention paid to differences between CHW-led interventions in low- and middle-income countries (LMICs) and high-income countries (HICs).**Methods**: A scoping review was performed using six databases with dates ranging from 1978 to 2019. Comprehensive definitions and search terms were established for ‘Community Health Workers’ and ‘breast cancer screening’, and studies were extracted using the World Bank definition of LMIC. Screening and data extraction were protocolized using multiple independent reviewers. Chi-square test of independence was used for statistical analysis of the incidence of themes in HICs and LMICs.**Results**: Of the 1,551 papers screened, 33 were included based on inclusion and exclusion criteria. Study locations included the United States (n=27), Bangladesh (n=1), Peru (n=1), Malawi (n=2), Rwanda (n=1), and South Africa (n=1). Three primary roles for CHWs in breast cancer screening were identified: education (n=30), direct assistance or performance of breast cancer screening (n=7), and navigational services (n=6). In these roles, CHWs improved rates of breast cancer screening (n=23) and overall community member knowledge (n=21). Two studies performed cost-analyses of CHW-led interventions.**Conclusion**: This review extends our understanding of CHW effectiveness to breast cancer screening. It illustrates how CHW involvement in screening programs can have a significant impact in LMICs and HICs, and highlights the three CHW roles of education, direct performance of screening, and navigational services that emerge as useful pillars around which governments and NGOs can design effective programs in this area.

## Background

Breast cancer is the leading cause of cancer among women globally [1,2] According to the World Cancer Research Fund, the number of new breast cancer cases in 2012 was approximately 1.7 million globally; in 2019, this number reached 2 million [3]. Incidence and prevalence rates vary from country to country, but prevalence is higher in developed nations [3]. Recent years, however, have seen an upward trend in breast cancer prevalence regardless of a country’s development status. In fact, the World Health Organization (WHO) reports that 50% of breast cancers now occur in the developing world [4,5]. Irrespective of these trends, breast cancer imposes a disproportionate burden on developing countries [6]. The 2016 Global Burden of Disease Study highlights this disparity, demonstrating a correlation between the human development index (HDI) of a country and the breast cancer mortality-to-incidence ratio, where the lowest ratio (and highest relative mortality) is associated with low HDI countries while higher ratios are found in very high HDI countries [7]. This disproportionate burden calls for more effective and scalable approaches to addressing the problem [6,8].

Even within the United States (US) and other high-income countries (HICs), breast cancer deaths are significantly divided by race and ethnicity [9]. For example, non-Hispanic black women in the US are more likely to die from breast cancer across all age groups than any other race or ethnicity [10]. Based on data from 2013 to 2017, the breast cancer death rate for American black women is 40% higher when compared to the rate for white women, despite black women having a lower overall incidence of breast cancer [11,12]. While this discrepancy may be explained in part by the higher prevalence of triple-negative breast cancer among black women [13], there are also larger social and systemic factors, such as delayed access to healthcare for both screening and treatment, that impact breast cancer survival [9–11].

In response to these startling figures and to the anticipated shortage of healthcare workers worldwide, healthcare systems around the globe have deployed Community Health Workers (CHWs) to provide an integrated approach to breast cancer screening [14]. CHWs are lay members of the community trained to ‘provide support and assistance to communities, families, and individuals with preventative health measures and gain access to appropriate curative health and social services [15].’ These individuals serve as health and cultural advocates; they are from the communities they serve, have a shared cultural understanding, and speak a common language with their constituents. This skill set empowers CHWs with the unique opportunity to bridge the gap between local health systems and their communities and to act as agents for social change [13]. In low- and middle-income countries (LMICs), they often fill the role of front line primary care personnel by providing cancer screening, low-risk interventions, and medication distribution [15].

CHWs have demonstrated their value by strengthening primary care services in complex health systems, working to combat both communicable diseases and non-communicable diseases (NCDs), including diabetes, hypertension, and tobacco cessation [16]. Their beneficial role in LMICs has been documented in the context of cervical and breast cancer screening, where CHW educators and coordinators improved screening [17,18]. It has also been documented in the US, where increased training and resources for CHWs improved mammography rates [19]. This backdrop spurred the WHO to develop guidelines focused on the optimization of CHW programs in diverse healthcare arenas [15]. The studies used by the WHO to create these guidelines demonstrated the positive benefits of CHW interventions on overall cancer screening rates and knowledge [15]. However, only one study mentioned in this report discussed the potential for CHW to address cancer screening in LMICs specifically, and it “did not provide evidence of CHW capacity [20].”

CHW effectiveness in these areas inspires the question of how CHW interventions can be leveraged in other contexts, such as that of breast cancer screening. Indeed, while reviews focused on CHWs within LMICs exist [18], there is a lack of literature that comprehensively reviews the role of CHWs in breast cancer screening across both HICs and LMICs. Viewed through the lens of global healthcare during the concurrent pandemics of systemic racism and COVID-19, this insight is particularly timely to better understand creative, community-driven approaches to address health disparities for under-served populations and populations with limited access to high quality care.

In light of this gap in the literature, this review paper aims to answer the following questions:

1. What are the roles for CHWs in breast cancer screening?
2. What are the impacts of CHWs when involved in breast cancer screening?
3. Do the roles of CHWs in breast cancer screening differ between LMICs and HICs?
4. Do these roles provide an appropriate and feasible avenue for future programs with CHWs and breast cancer screening? More specifically, are these programs economically feasible?
5. Once these questions are explored, how can the authors imagine a post-pandemic world in which CHWs contribute to healthcare systems that value and ensure justice and equity in allocation and distribution of resources to address breast cancer outcomes?

## Methods

### Review approach

A scoping review was conducted on the role of CHWs in breast cancer screening. Scoping reviews identify and characterize existing literature about a topic of interest to present an overview of a diverse and broad body of evidence [21]. This method allows for examination of the existing literature to present current evidence on ways in which CHWs are used in breast cancer screening across varied geographic areas and heterogenous cultural contexts.

### Search strategy and selection criteria

Six electronic bibliographic databases (PubMed, Scopus, Web of Science, Global Health, Cochrane Breast Cancer, and ProQuest) were systematically searched to capture relevant articles from medical sciences, public health, and global health. These databases were searched with individualized search strings that included CHWs and equivalent terms, in addition to terms focused on breast cancer and breast cancer screening (Appendix A). The search criteria included date (January 1978 to August 2019), language (English and French), and type of paper (randomized controlled trials, mixed method approaches, observational cohort studies, government policies/guidelines and unpublished studies found in grey literature). All eligible papers were imported into the online platform Covidence, which was used for title and abstract screening and subsequent full text review.

### Inclusion and exclusion criteria

Papers were included if [1] CHWs, whether paid or unpaid, were the primary means of breast cancer screening and/or breast cancer management, including prevention and treatment and [2], if the study explicitly stated that the objective or aim was the screening of breast cancer by CHWs at the community level. In our study, CHWs were defined as lay people who do not hold a clinical license and have duties among the community, including activities such as health promotion, prevention, and delivery capacity. To be included, articles had to describe CHWs as a distinct occupation from other associated healthcare workers [22]. They were differentiated from Patient Navigators (PNs), who were assigned specific patients, had duties other than that of CHWs, and were a role/function rather than the distinct occupation of a CHW [23].

Papers were excluded if [1] the primary focus was on health care professionals other than CHWs (doctors, nurses, medical students, PNs, and other allied healthcare professionals), [2] it lacked a community-based approach to healthcare delivery, [3] it focused on diseases or conditions other than breast cancer screening, prevention, treatment, and control, [4] it was not an original, full text, research study, including commentaries, letters, opinion pieces, study protocols, systematic reviews, and conference proceedings with only an abstract available.

### Study review and data analysis

For title and abstract screening, each paper was assessed in Covidence by two independent reviewers utilizing the above criteria. Conflicts were resolved by a third reviewer. The full texts of eligible studies were retrieved and independently assessed by two reviewers, and conflicts were resolved via consensus between all three reviewers. From the included studies, the study team extracted information regarding paper intervention, population characteristics, outcomes, content, limitations, the definition of CHWs, and the roles that they played in breast cancer screening (Appendix B). Key concepts from the papers were compiled for thematic analysis. Themes were analyzed for evidence of successful deployment of CHWs to address breast cancer screening and identified core attributes that contributed to the effectiveness of CHW-led interventions in their communities. Fisher’s Exact Test was used for statistical analysis of the incidence of these themes in HICs and LMICs. In this paper, LMICs will be defined using the World Bank criteria of GNI per capita of less than 12,375 USD and HICs above that value [24].

### Quality assessment

As this study represents a scoping review, a quality assessment was not conducted; this step is not traditionally included as part of this methodology [21]. As described by McColl et al. (2009), ‘the emphasis of a scoping study is on comprehensive coverage, rather than on a particular standard of evidence,’ so as to describe research activity and present existing literature about a topic [25]. While a formal quality assessment was not conducted for the reasons stated above, the authors of this study nonetheless sought to establish baseline quality parameters and only included papers that clearly identified their research goals, utilized appropriate methodologies and study design, and presented conclusions that matched the stated results.

## Results

### Search results

**Figure 1.**
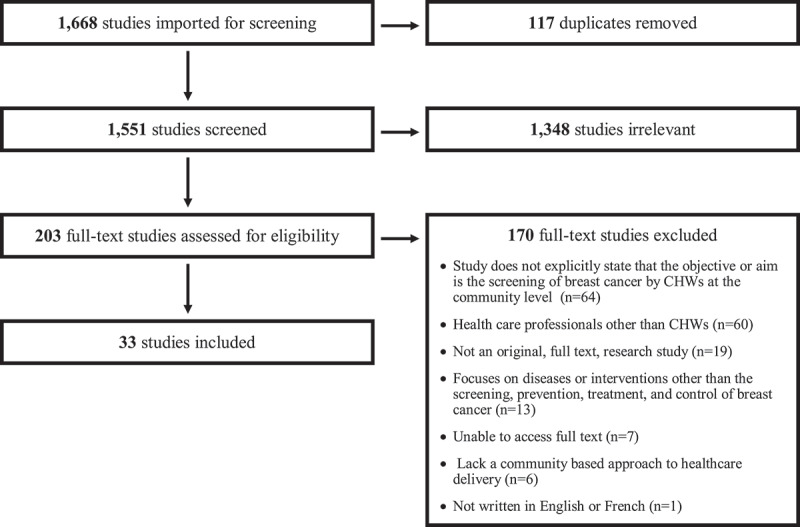
PRISMA flowchart of the included and excluded publications

The initial database search yielded a total of 1,668 papers. From that, 117 duplicates were removed leaving a total of 1,551 papers. After the title and abstract screening utilizing the inclusion and exclusion criteria above, 203 papers advanced to the full-text screening phase. The full text articles were then evaluated utilizing the same inclusion and exclusion criteria. This resulted in the exclusion of 170 papers, yielding 33 peer-reviewed papers examined for this review paper (Figure 1).

### Study characteristics

**Figure 2.**
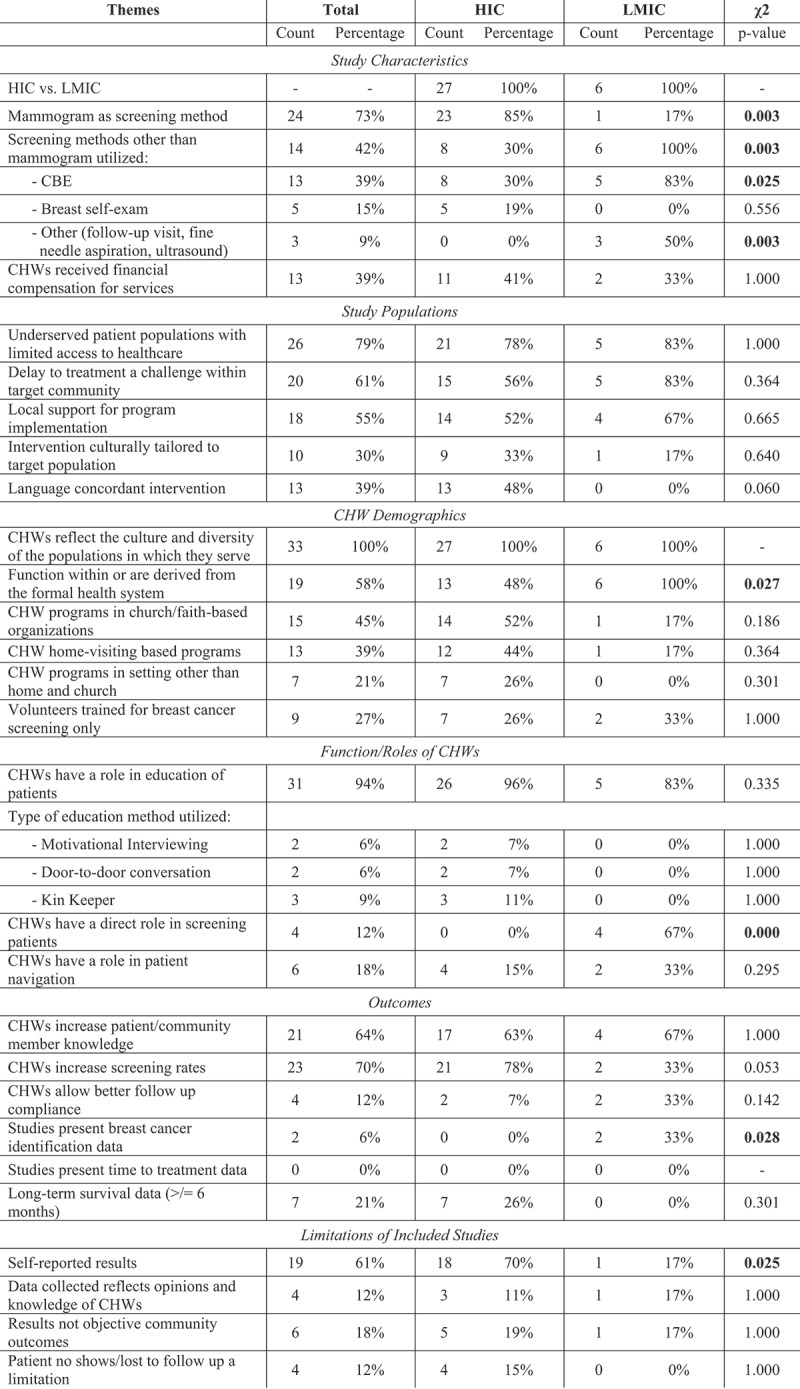
Themes were extracted and compiled from included publications to allow for examination of CHW deployment to address breast cancer screening. Fisher’s Exact Test was used for statistical analysis of the incidence of these themes in HIC and LMIC

The articles included in this review were published between 1997 and 2019. Of these, 27 studies were located in the US (n = 27). Studies conducted outside of the US included Bangladesh (n = 1), Peru (n = 1), Malawi (n = 2), Rwanda (n = 1), and South Africa (n = 1), all of which are considered LMICs. Within these papers, CHWs were diversely defined. A majority of the studies used a variation of CHW, Lay Health Advisor, or Community Health Representative (n = 22). Other definitions utilized more specific terms, such as *Promatoras* (n = 5), Breast Health Workers (n = 2), Neighborhood Assistants (n = 1), and Certified Midwives with roles similar to that of a CHW (n = 1). Henceforth, the term ‘CHW’ will be used to encompass all designations listed within Appendix B. CHWs were paid (n = 10) or received a stipend (n = 3) in 13 of the publications. Across all studies, breast cancer screening methods included screening mammograms (n = 24), clinical breast exams (CBE) (n = 13), or a mixed methods approach that incorporated both techniques (n = 8). There was a statistically significant difference between HICs and LMICs in their use of mammography as the screening method (HIC: 85%, LMIC: 17%, p-value: 0.003), versus other screening methods (HIC: 30%, LMIC: 100%, p-value: 0.003), particularly the use of CBE (HIC: 30%, LMIC: 83%, p-value: 0.025). All included themes and statistics can be reviewed in Figure 2.

### Study populations

Approximately 79% of the studies (n = 26) focused on underserved populations with limited access to healthcare. Of these studies, 21 were within HICs. Delay to treatment (n = 20), presentation to the formal healthcare system with advanced stages of disease, and decreased likelihood of receiving treatment were identified as problems within the target population of 20 studies. Local support for program implementation was seen in many of the studies (n = 18) including local, national, and international stakeholder involvement, health department/health center collaboration, and support from churches, academic centers or non-profit organizations (NGOs). 10 of the interventions were culturally tailored to their target population. Interventions were delivered in the primary language of the participants (Vietnamese, Spanish, Arabic, Chinese, Korean, Navajo) and implemented by fluent speakers in 13 studies.

### CHW demographics

The CHWs across all studies (n = 33) reflected the populations within which they served. Some of the CHW programs functioned within or were derived from the formal health system (n = 19); this broadly included CHW programs established and managed by health systems or non-governmental organizations (NGOs), and programs based at healthcare centers. It also encompassed CHWs trained by health systems or connected to specific health centers to which they referred patients. CHWs in LMICs were significantly more likely to be derived from the formal health system (HIC: 48%, LMIC: 100%, p-value: 0.027). These programs often functioned in diverse settings, including faith- or church- based programming (n = 15), home-visit based programming (n = 13), and outreach at health fairs, workplaces, schools, local businesses, or other venues (n = 7). While some of the interventions utilized already established CHWs, others trained new CHWs for the implementation of their breast cancer programming or with a specific focus on breast cancer screening and prevention (n = 9).

### Function/roles of CHWs

Across the 33 studies, CHWs served in a variety of roles. This review specifically identified three primary roles for CHWs discussed in the literature: education about breast cancer and the importance of screening (n = 31), direct assistance in or performance of breast cancer screening (n = 7), and navigational services (n = 6).

Education was by far the most prevalent role that CHWs performed. CHWs provided information about general breast health, breast cancer signs and symptoms, breast cancer screening methods per national guidelines, as well as community-specific breast cancer resources. Education occurred in many settings, such as homes, churches, and community centers. Teaching methods utilized by CHWs in these settings include Motivational Interviewing (MI, n = 2), door-to-door conversations (n = 2), and Kin Keeper home visits (n = 3). Used by Brandford et al. (2019), MI is a technique that emphasizes ‘communication skills like reflective listening’ to promote healthy behavior change [26]. In this study, CHWs were able to effectively implement MI strategies, and felt more confident talking to community members about breast cancer screening. Another educational method utilized was The Kin Keeper model, which teaches women in family units. As Williams et al. (2009) describes, this model assumes that ‘female family relationships are synergistic such that empowerment or self-efficacy education for individual women could engage other female family members [27].’

Direct assistance in breast cancer screening was defined as active participation by a CHW in a screening procedure, such as a CBE, or assistance with a procedure performed by another medical provider. This assistance could be done in a clinic or in the community. Using this definition, four studies met criteria. CHWs performed CBEs in three of these studies. In the fourth study, they assisted nurses who performed CBEs in the clinic. Of note, Gutnik et al. (2016) found that CBEs performed by CHWs in Malawi had a sensitivity of 94%, specificity of 58%, positive predictive value of 48%, and were comparable to CBEs performed by physicians in the study [28]. Only papers within LMICs utilized CHWs directly in breast cancer screening (HIC: 0%, LMIC: 67%, p-value: <0.001).

Finally, CHWs assisted constituents in navigating complex health care systems (n = 6). Examples of this role included screening recruitment, scheduling initial appointments with primary care providers or radiology services for mammograms, following up with screening results, and scheduling treatment appointments if a woman was diagnosed with breast cancer. The majority of the studies that employed navigation were in the US, with the exception of one study in South Africa and another in Bangladesh. In the study by Hunt et al. (2017), these CHWs provided extensive navigational services in Chicago that did not stop until a patient completed screening, treatment, or a patient elected to discontinue the services [29].

### Outcomes

CHW-based interventions were associated with an increase in overall community member knowledge in 63% of studies (n = 21). This knowledge included general breast health, breast cancer information, proper breast cancer screening methods, and signs and symptoms of breast cancer. Only one study reported no change in community member knowledge after the CHW intervention [30]. This study implemented classroom sessions taught by CHWs for Korean American women, combined with follow-up counseling and navigational assistance. The intervention improved rates of mammograms and CBEs, but not cancer knowledge or perceptions of cancer.

CHW-led interventions were associated with an increase in breast-cancer screening rates in 70% of papers (n = 23). This included increased numbers of screening mammograms, screening appointments with providers, and CBEs. Four studies reported that CHWs increased cancer screening follow-up compliance. One of these studies out of Malawi reported an increase in compliance when CHWs were taught to perform CBEs specifically. In addition to screening rates, they found that there was an increase in community breast cancer knowledge overall, as CHWs educated their communities while performing CBEs [28]. Additionally, two studies reported breast cancer identification data; for example, Duggan et al. (2017) screened 13,500 women in Peru with CBEs performed by midwives and identified 321 women with breast abnormalities [31]. Each woman received fine needle aspiration (FNA) of the lesion, of which 10 women were identified to have breast cancer. Additionally, the 2015 study by Chowdhury et al. in Bangladesh identified a case of stage III breast cancer in the CHW-led intervention group [32]. Of note, these publications demonstrating identification data were both in LMICs, which was significant when compared to HICs (HIC: 0%, LMIC: 33%, p-value: 0.028). Despite this, neither of these studies used a control, making it difficult to truly assess the impact of CHWs on these results.

Only nine studies contained long term survival data >/= 6 months, and just three of these contain long term survival data >/= one year. Ford et al. (2014) mentioned three year follow-up for patients, but the data was not included in the publication [33]. No included studies presented time-to-treatment data related to CHW-led interventions, which is a serious limitation of this approach.

### Limitations of included studies

In 20 of the included papers, the results were self-reported by either participants or CHWs. These results included self-reported knowledge of breast cancer screening, self-reported adherence to breast cancer screening recommendations, and self-evaluation of CHW activities. Self-reported results were predominantly found in HICs (HIC: 67%, LMIC: 17%, p-value: 0.025). In addition, the data collected in four studies reflected the opinions and knowledge of CHWs: their confidence, satisfaction, and impact. Published outcomes were not objective community outcomes in six papers and four studies specifically mentioned patients being lost to follow-up as a concern.

### Financial considerations

Two studies, both from the US, reported financial data and performed a formal cost analysis of a CHW-led intervention. In Phoenix, Arizona, Larkey et al. (2002) implemented CHW-taught cancer screening and prevention classes delivered individually (IND) and in a social support group (SSG) [34]. Costs analyzed included participant transportation, cancer screening, salaries, and supplies. The authors noted the cost to achieve one cancer screening in the study population ranged from 263 USD to 517 USD in the SSG 475 arm and from 862 USD to 1716 USD for the IND arm. Cancer screening included breast, cervical, and colorectal, and results were not stratified based on type of cancer. In Schuster et al. (2015), incremental cost-effectiveness ratios (ICERs) were determined, which represented the additional costs of the interventional arm of the study compared to the control arm [35]. The authors determined that their CHW-led intervention added an additional 236 USD to breast cancer screening costs compared to the control group, which the authors attribute largely to salaries of the CHWs. There were no formal cost analyses performed for studies in LMICs.

### Methodological limitations

As there is no fixed definition for CHWs, some exclusions from this paper may be contested. In addition, broad conclusions may be difficult to draw based on the contextually specific nature of the projects discussed in these studies. Although the themes in this paper were agreed upon by all authors, it is possible that additional themes could have been identified based on alternative interpretations of the literature. In addition, a quality assessment was not conducted, as is traditional for scoping reviews. This choice enabled the authors to include a greater variety of studies than would have been possible in a systematic review.

## Discussion

This scoping review assessed the roles that CHWs can play in breast cancer screening across geographically and culturally heterogeneous settings, and how these roles differ in HICs versus LMICs. Drawing on the data analyzed, this review sought to determine how CHW involvement in breast cancer screening efforts affect outcomes. Given the rising global burden of cancer and the paucity of survival data, it is reasonable to ask how CHWs can be successfully integrated into highly functioning health systems to improve quality of care for breast cancer patients in low resource settings of HICs and LMICs. Further improvements in knowledge will contribute to the literature and help driving evidence-based, feasible CHW screening and education programs aimed at improving breast cancer outcomes.

Three major areas were identified as benefiting from CHW involvement: education, screening, and navigational services. Lack of information and understanding of breast cancer have been identified as contributing to differences in timely follow-up for low‐income, ethnically diverse women [36], who report a greater need for information delivered in culturally appropriate formats [37]. CHWs were particularly effective as peer educators for their community members with over half of the studies demonstrating a significant increase in patients’ understanding of breast cancer and the need for breast cancer screening. One posited reason for this success is that CHW-led interventions were likely to be linguistically and culturally concordant with the patient community. As community representatives and advocates, CHWs are able to make stronger connections and provide advice and information relevant to specific populations facing barriers in accessing healthcare [36,37]. In 2015, Daly and Olopade argued that moving the needle for disparities in breast cancer starts with emphasis on rectifying the lack of education on and understanding of breast cancer genomics, genetic counseling and the BRCA genes [9,38,39]. CHWs integrated in breast cancer care may benefit from training focused on educating their patients on these topics, as this knowledge will equip communities with the knowledge to advocate for high quality precision-based care.

While CHWs were found to be effective educators across many studies, the forum through which their message is best shared remains unclear. The studies employed a diverse array of venues and methodologies through which CHWs disseminated health literacy information. These methods included motivational interviewing and the KinKeeper model, which both led to improvement in overall breast cancer knowledge. Additionally, Larkey et al. demonstrated that there was no significant difference between one-on-one or group-based CHW-led education sessions [34]. Based on financial analysis and other studies presented in this review, group-based CHW interventions can be an efficient and less expensive method for improving community education compared to individualized interventions [34].

CHWs were also effective at providing breast cancer screening directly to patients through CBEs in LMICs. In the study by Gutnik et al. (2016), with appropriate training, CBEs performed by CHWs had a high negative predictive value and were comparable in sensitivity and specificity to the physician providers [28]. Of the studies in LMICs, CBEs were the predominant form of screening, as compared to HICs where mammograms were primarily utilized. This review also found that CHWs were only used directly for screening in LMICs. These differences were all statistically significant and may indicate limited access to mammography services in LMICs. This serves as a call to action for investment in breast cancer diagnostic and treatment services in HICs and LMICs. While evidence of the influence of CHWs on timely detection of breast cancer is presently limited, identifying tumors at an early stage to improve survival requires accessibility to the basic standard of care for breast cancer detection, which includes up-to-date mammography equipment and well-trained mammographers [40].

CHWs fulfilled a variety of navigational responsibilities that were beneficial to the community and oftentimes, had overlap with navigational services traditionally performed by patient navigators. In multiple studies, CHWs proved to be a natural bridge between the community and the formal healthcare system, from providing mammography and primary care provider (PCP) referrals to a longer-term relationship with one participant from referral to diagnosis and treatment. In the study by Hunt et al. (2017), CHWs were shown to be crucial members in establishing a breast cancer program in the community and helping women in the community understand the need for screening, while also assisting women in receiving the necessary healthcare services [29]. Additionally, having CHWs as both educators and navigators in this program provided greater continuity of care for these patients. Through the synergistic efforts of both successful education and navigational interventions, this study saw a significant increase in mammography services in a vulnerable patient population [29]. Having navigational assistance improves community outcomes and CHWs can help fulfill that need if there is a shortage of PNs. This service may be particularly beneficial in settings where CHWs function within or are derived from the formal health system, which this paper found predominantly in LMICs. Notably, as the COVID-19 pandemic has increased barriers to healthcare across the globe, telehealth has emerged as an innovative approach to narrowing gaps in accessing care. In the CHW context, there are significant opportunities for telehealth to play a role in synergistically improving access to education and screening, and particularly for navigational services. Future programs may be able to create multidisciplinary teams where CHWs focus on education and screening, while PNs fulfill the navigational responsibilities. When reimagining the landscape of breast cancer care, Daly and Olopade emphasize the importance of PNs and CHWs in helping patients overcome logistical barriers to accessing services, but also caution that they cannot operate in a vacuum and must come along with broader system-based changes that integrate these services.

Furthermore, it is important to discuss not only the possibility of including CHWs in diverse roles, but also if their inclusion is appropriate and effective. The feasibility of including CHWs in breast cancer screening can be considered in financial terms, as well as through a logistical lens. Two studies noted that the inclusion of CHWs in breast cancer screening projects added hundreds of dollars to the cost of their work. This figure is notable, as this increased cost could be prohibitive for programs with fewer financial resources. However, this sum should be considered within context, as the role of a CHW is not always a paid position around the globe and, therefore, should not discourage architects of breast cancer screening programs from including CHWs in their work. In addition, as both studies were performed in the US, the higher costs may not actually be representative of CHW program costs in LMICs and other HICs. While this study has demonstrated that CHW involvement in a wide variety of roles in breast cancer screening is possible, this idea should be weighed alongside the other demands that are placed on CHWs and the diverse gaps that they are called upon to fill within global health systems. Future research on how to prioritize or specialize CHW time and training related to breast cancer screening versus their other roles would be beneficial for overall CHW program design.

Notably, this review revealed that there is a limited pool of studies that have explored the use of CHWs in the context of breast cancer in LMICs. Of the 33 studies included, only six were conducted in LMICs. While this small sample size limits applicability, the six articles convincingly highlight CHWs effectiveness in these contexts. In all of the studies within LMICs, the use of CHWs in breast cancer screening was positively correlated with targeted outcomes [28,31,32,41–43]. In addition, exogenous benefits were also reported. Pace et al. (2018) found a more than 10-fold increase in reports of CHWs engaging with community members about health concerns as a result of incorporating CBEs into their work [42]. This information suggests that immersive engagement with community members by CHWs about breast health leads to more conversations about general health. Additional research on the role of CHWs in providing breast cancer education, screening, recruitment for screening, and navigation in LMICs would greatly contribute to understanding how these interventions can be most effectively tailored to the needs of specific populations. A discussion of these ideas, however, would be incomplete without emphasizing the paucity of high-quality studies about CHWs and breast cancer screening, particularly in LMICs. The lack of rigorous data has significant implications for accurately assessing the impact of CHW activity in these settings. This study, therefore, underscores the necessity of investing in and conducting high quality studies on this topic in LMICs, which can inform evidence on the impact of this approach. For the studies performed in HICs, the majority focused on underserved populations with limited access to the formal healthcare system. While systemic and contextual differences exist, these populations share important characteristics with target populations for CHWs in LMICs, particularly with respect to disparities in accessing breast cancer screening. These similarities are reflected in the analysis conducted within this paper, which identified few statistically significant differences between study populations, CHW demographics, and the roles of CHWs across all included studies. The identified problems facing the populations in HICs – treatment delay, healthcare system involvement at advanced stages, and decreased levels of treatment – are largely the same risk factors that make CHWs uniquely suited to address similar health disparities in LMICs. Given these similarities, until further research investigates this topic in LMICs, it may be appropriate to extrapolate the data from communities in HICs experiencing health disparities. While these groups are not a perfect comparison, they can serve as an indirect guide for communities looking to utilize CHWs for breast cancer screening. More broadly, gaps in breast cancer literature and breast cancer care extend beyond LMICs, with a notable lack of focus on minority populations [44]. The COVID-19 pandemic has elucidated the systemic racism embedded in healthcare, and it is essential that we now face this inequity head on when addressing the glaring disparities in breast cancer health outcomes.

While our research demonstrates that CHWs are effective in diverse roles in breast cancer screening, the long-term impact of these roles is not clear. CHWs have been lauded as the answer to addressing health disparities in marginalized communities; however, our data does not indicate that these interventions significantly decreased morbidity or mortality from breast cancer in these communities. The primary driver of this limitation is likely due to a lack of follow-up from these studies and the significant prevalence of self-reported data within HICs, rather than an overall lack of long-term efficacy. Ultimately, further studies analyzing the long-term effects of CHW-led interventions, such as overall morbidity and mortality, are needed in the literature in order to adequately address this crucial outcome. Recommending specific interventions is challenging, given the broad scope of the CHW role within diverse contexts. Future investigators should focus on methods, such as the step wedge approach, that incorporate tracking of long-term outcomes to allow researchers to better isolate which interventions are making significant impacts on improving population health. Additionally, it is impossible to advocate for larger scale research targeting CHWs without championing broader delivery system reform within which these CHWs operate. Solutions proposed by Daly and Olopade include accountable care organizations (ACOs) targeted towards a specific patient population which is responsible for their care and health outcomes and an emphasis on precision medicine for all.

Now more than ever, with healthcare systems pushed to their limits from the novel COVID-19 pandemic, communities need unique and creative solutions to address health disparities that are both equitable and social-justice oriented. First and foremost, to effectively improve breast cancer outcomes, CHW programs need an effective healthcare system behind them. Investment in CHW programs as a part of broader strengthening of global health systems is imperative to close the gap in patient outcomes. With the robust support of a well-established health infrastructure, CHWs can be advocates and leaders within the communities they represent, which are so often neglected by current healthcare systems. This focus on community-driven care amplifies the voices of those disparaged, which are often low-income communities and communities of color. When CHWs are not only able to diagnose, but also help women access and navigate a system that offers the most appropriate and efficacious treatment available, we will be able to see meaningful improvements in breast cancer disparities and outcomes.

## Conclusion

While prior reviews have demonstrated CHW effectiveness in providing front line primary care, this review has extended our understanding of that effectiveness to breast cancer screening. It highlights how CHWs can and should be included in breast cancer screening programs, and how CHW involvement in such programs can improve breast cancer impacts in both LMICs and HICs. The current literature addresses multiple roles for CHWs in breast cancer screening. Trained CHWs can be effective educators, teaching the community about breast health and guidelines for proper breast cancer screening. CHWs can be effective members of a health care team dedicated to breast cancer screening, in which they perform CBEs or assist other medical providers in screening procedures. They can also successfully perform specific navigational roles, including assistance with scheduling screening appointments and with any necessary follow-up care. All these roles help to increase community members’ knowledge of breast cancer screening and can improve rates of appropriate screening methods, such as CBEs and mammography rates. As a result, education, direct performance of screening, and navigational services emerge as useful pillars around which governments and NGOs can design effective programs that incorporate CHW-based breast cancer screening. We hope that in providing a culturally competent and ethical framework for CHWs to participate in this global conversation on breast cancer, more countries will be able to develop programs that utilize their expertise.

## Paper context

This paper defines specific roles for CHWs in breast cancer screening supported by the existing literature. We hope that governments and NGOs working with CHWs can use the results to train and support CHWs in the most effective and equitable manner, both for the CHWs and the communities they serve. This paper also suggests future research avenues, including long-term outcomes and survival data needed in order to continue studying this important topic.

## Appendix 1

**Appendix A.** Specific search strings for the six electronic databases (PubMed, Scopus, Web of Science, Global Health, Cochrane Breast Cancer, and ProQuest) included in this review

PubMed	((((“Allied health personnel” OR “Auxiliary health worker*” OR “Barefoot doctor*” OR “Basic health worker*” OR “Community assistant*” OR “Community drug distributor*” OR “Community health representative*” OR “Community health advocate*” OR “Community health agent*” OR “Community health aide*” OR “Community health promoter*” OR “Community health volunteer*” OR “Community health worker*” OR “Community mobilizer*” OR “Community nutrition worker*” OR “Community resource person” OR “Community reproductive health worker*” OR “Community support worker*” OR “Community volunteer*” OR “Community-based worker*” OR “Female community health volunteer*” OR “Female multipurpose health worker*” OR “Health and nutrition worker*” OR “Health service assistant*” OR “Health surveillance assistant*” OR “Health promoter*” OR “Home health aide*” OR “Lady health worker*” OR “Lay health visitor*” OR “Lay health worker*” OR “Link worker*” OR “Maternal and child health promotion worker*” OR “Maternal and child health worker*” OR “Mental health worker*” OR “Mother coordinator*” OR “Nutrition volunteer*” OR “Nutrition worker*” OR “Outreach educator*” OR “Paramedical worker*” OR “Peer volunteer*” OR “Postnatal support worker*” OR “Rural health motivator*” OR “Rural health worker*” OR “Village drug-kit manager*” OR “Village Health Guide” OR “Village health helper*” OR “Village health worker*” OR “Village health team*” OR “Voluntary worker*” OR “Women group leader*”)) **AND** (“Breast cancer“[Mesh] OR “breast cancer” OR “breast lump” OR “breast exam”))
Scopus	((TITLE-ABS-KEY (“Allied health personnel” OR “Auxiliary health worker*” OR “Barefoot doctor*” OR “Basic health worker*” OR “Community assistant*” OR “Community drug distributor*” OR “Community health representative*” OR “Community health advocate*” OR “Community health agent*” OR “Community health aide*” OR “Community health promoter*” OR “Community health volunteer*” OR “Community health worker*” OR “Community mobilizer*”) OR TITLE-ABS-KEY (“Community nutrition worker*” OR “Community resource person” OR “Community reproductive health worker*” OR “Community support worker*” OR “Community volunteer*” OR “Community-based worker*” OR “Female community health volunteer*”) OR TITLE-ABS-KEY (“Female multipurpose health worker*” OR “Health and nutrition worker*” OR “Health service assistant*” OR “Health surveillance assistant*” OR “Health promoter*” OR “Home health aide*” OR “Lady health worker*” OR “Lay health visitor*”) OR TITLE-ABS-KEY (“Lay health worker*” OR “Link worker*” OR “Maternal and child health promotion worker*” OR “Maternal and child health worker*” OR “Mental health worker*” OR “Mother coordinator*”) OR TITLE-ABS-KEY (“Nutrition volunteer*” OR “Nutrition worker*” OR “Outreach educator*” OR “Paramedical worker*” OR “Peer volunteer*” OR “Postnatal support worker*” OR “Rural health motivator*”) OR TITLE-ABS-KEY (“Rural health worker*” OR “Village drug-kit manager*” OR “Village Health Guide” OR “Village health helper*” OR “Village health worker*” OR “Village health team*” OR “Voluntary worker*” OR “Women group leader*”)) AND PUBYEAR > 1977) **AND** (TITLE-ABS-KEY (“breast cancer” OR “breast carcinoma” OR “breast lump” OR “breast exam”) AND PUBYEAR > 1977)
Web of Science	(TS=(“Allied health personnel”) OR TS=(“Auxiliary health worker*”) OR TS=(“barefoot doctor*”) OR TS=(“Basic health worker*”) OR TS=(“Community assistant*”) OR TS=(“Community drug distributor*”) OR TS=(“Community health representative*”)OR TS=(“Community health advocate*”) OR TS=(“Community health agent*”) OR TS=(“Community health aide*”) OR TS=(“Community health promoter*”) OR TS=(“Community health volunteer*”) OR TS=(“Community health worker*”) OR TS=(“Community mobilizer*”) OR TS=(“Community nutrition worker*”) OR TS=(“Community resource person*”) OR TS=(“Community reproductive health worker*”) OR TS=(“Community support worker*”) OR TS=(“community volunteer*”) OR TS=(“Community-based worker*”) OR TS=(“Female community health volunteer*”) OR TS=(“Female multipurpose health worker*”) OR TS=(“Health and nutrition worker*”) OR TS=(“Health service assistant*”) OR TS=(“Health surveillance assistant*”) OR TS=(“health promoter*”) OR TS=(“Home health aide*”) OR TS=(“Lady health worker*”) OR TS=(“Lay health visitor*”) OR TS=(“Lay health worker*”) OR TS=(“Link worker*”) OR TS=(“Maternal and child health promotion worker*”) OR TS=(“Maternal and child health worker*”) OR TS=(“Mental health worker*”) OR TS=(“Mother coordinator*”) OR TS=(“Nutrition volunteer*”) OR TS=(“Nutrition worker*”) OR TS=(“Outreach educator*”) OR TS=(“Paramedical worker*”) OR TS=(“Peer volunteer*”) OR TS=(“Postnatal support worker*”) OR TS=(“Rural health motivator*”) OR TS=(“Rural health worker*”) OR TS=(“Village drug-kit manager*”) OR TS=(“Village Health Guide”) OR TS=(“Village health helper*”) OR TS=(“Village health worker*”) OR TS=(“Village health team*”) OR TS=(“voluntary worker*”) OR TS=(“Women group leader*”)) **AND** (TS=(“breast cancer*”) OR TS=(“breast carcinoma*”) OR TS=(“breast lump*”) OR TS=(“breast exam*”))
Global Health	1. (allied health personnel or Auxiliary health worker* or barefoot doctor* or basic health worker* or community assistant* or community drug distributor* or community health representative* or community health advocate* or community health agent* or Community health aide* or Community health promoter* or Community health volunteer* or Community health worker* or Community mobilizer* or Community nutrition worker* or Community resource person or Community reproductive health worker* or Community support worker* or Community volunteer* or Community-based worker* or Female community health volunteer* or Female multipurpose health worker* or (Health and nutrition worker*) or Health service assistant* or Health surveillance assistant* or Health promoter* or Home health aide* or Lady health worker* or Lay health visitor* or Lay health worker* or Link worker* or (Maternal and child health promotion worker*) or (Maternal and child health worker*) or Mental health worker* or Mother coordinator* or Nutrition volunteer or Nutrition worker* or Outreach educator* or Paramedical worker* or Peer volunteer* or Postnatal support worker* or Rural health motivator* or Rural health worker* or Village drug-kit manager* or Village Health Guide or Village health helper* or Village health worker* or village health team* or Voluntary worker* or Women group leader*).**af**. 2. (breast cancer or breast carcinoma or breast lump or breast exam).**af**. 3. 1 AND 2
Cochrane Breast Cancer	#1 MeSH descriptor: [Community Health Workers] explode all trees #2 MeSH descriptor: [Health Personnel] explode all trees #3 MeSH descriptor: (health worker* or health care worker* or health professional or health personnel or doctor* or nurse* or physician*) #4 MeSH descriptor: #1 or #2 or #3 #5 MeSH descriptor: [Health Education] explode all trees #6 MeSH descriptor: [Education, Continuing] explode all trees #7 MeSH descriptor: [Education, Medical] explode all trees #8 MeSH descriptor: [Education, Nursing] explode all trees #9 MeSH descriptor: (train* or training or education or curriculum or teaching or learning or staff development or medicine) #10 MeSH descriptor: #5 or #6 or #7 or #8 or #9 #11 MeSH descriptor: #4 and #10 #12 MeSH descriptor: [Health Knowledge, Attitudes, Practice] explode all trees #13 MeSH descriptor: #11 or #12 #14 MeSH descriptor: [Physical Examination] explode all trees #15 MeSH descriptor: ((physical or clinical breast or clinical or breast) adj1 exam*) #16 MeSH descriptor: clinical breast examination #17 MeSH descriptor: [Mass Screening] explode all trees #18 MeSH descriptor: #14 or #15 or #16 or #17 #19 MeSH descriptor: [Breast Neoplasms] explode all trees #20 MeSH descriptor: breast near neoplasm* #21 MeSH descriptor: breast near carcinom* #22 MeSH descriptor: breast near cancer* #23 MeSH descriptor: breast near tumour* #24 MeSH descriptor: breast near tumor* #25 MeSH descriptor: breast near malignan* #26 MeSH descriptor: #19 or #20 or #21 or #22 or #23 or #24 or #25 #27 MeSH descriptor: #13 and #18 and #26 Search string based on Sayed et al. (2017)^a^
ProQuest	*Included databases*: Health & Medical Collection, Nursing & Allied Health Database, Public Health Database, Social Science Database, Sociology Database *Document type included*: Article, Dissertation/Thesis, Evidence Based Healthcare (“Allied health personnel” OR “Auxiliary health worker*” OR (“barefoot doctor” OR “barefoot doctors”) OR “Basic health worker*” OR “Community assistant*” OR “Community drug distributor*” OR “Community health representative*” OR “Community health advocate*” OR “Community health agent*” OR “Community health aide*” OR “Community health promoter*” OR “Community health volunteer*” OR “Community health worker*” OR “Community mobilizer*” OR “Community nutrition worker*” OR “Community resource person” OR “Community reproductive health worker*” OR “Community support worker*” OR (“community volunteer” OR “community volunteering” OR “community volunteerism” OR “community volunteers”) OR “Community-based worker*” OR “Female community health volunteer*” OR “Female multipurpose health worker*” OR “Health and nutrition worker*” OR “Health service assistant*” OR “Health surveillance assistant*” OR (“health promoters”) OR “Home health aide*” OR “Lady health worker*” OR “Lay health visitor*” OR “Lay health worker*” OR “Link worker*” OR “Maternal and child health promotion worker*” OR “Maternal and child health worker*” OR “Mental health worker*” OR “Mother coordinator*” OR “Nutrition volunteer*” OR “Nutrition worker*” OR “Outreach educator*” OR “Paramedical worker*” OR “Peer volunteer*” OR “Postnatal support worker*” OR “Rural health motivator*” OR “Rural health worker*” OR “Village drug-kit manager*” OR “Village Health Guide” OR “Village health helper*” OR “Village health worker*” OR “Village health team*” OR (“voluntary worker” OR “voluntary workers”) OR “Women group leader*”) **AND** (“breast cancer*” OR “breast carcinoma*” OR “breast lump*” OR “breast exam*”) AND pd(1977–2019)

^a^Sayed S, Ngugi A, Ochieng P, Mwenda AS, Salam RA. Training health workers in clinical breast examination for early detection of breast cancer in low‐ and middle‐income countries. *Cochrane Database Syst Rev*. 2017;2017[1]. doi:10.1002/14651858.CD012515

## Appendix B.

**Appendix B.** Specific data extracted from each individual paper included in this review to be used for analysis, including but not limited to summaries, methods, outcomes, and limitations

Author	Title of Paper	Publication Year	Journal	Volume	Issue	Country	Location (including Region/District)	Primary Aim of the Paper
Ahmed, N.U.; Haber, G.; Semenya, K.A.; Hargreaves, M.K.	Randomized controlled trial of mammography intervention in insured very low-income women	2010	Cancer Epidemiol. Biomarkers Prev.	19	7	United States	Tennessee	To determine if intervention with letter vs. letter with follow up by lay health workers will improve compliance with mammography screening
Bird, J.A.; McPhee, S.J.; Ha, N.-T.; Le, B.; Davis, T.; Jenkins, C.N.H.	Opening pathways to cancer screening for Vietnamese-American women: Lay health workers hold a key	1998	Prev. Med.	27	6	United States	San Francisco, California	To address breast and cervical cancer in the Vietnamese population in California by implementing preventive measures and education with lay health workers who speak the same language and are able to reach the population on a culterally relevant level.
Brandford, A.; Adegboyega, A.; Combs, B.; Hatcher, J.	Training Community Health Workers in motivational interviewing to promote cancer screening	2019	Health Promot. Pract.	20	2	United States	Kentucky	To describe the feasibility of training CHWs to deliver an Motivational Interviewing (MI) intervention to promote cancer screening in underserved populations.
Chowdhury, T.I.; Love, R.R.; Chowdhury, M.T.I.; Artif, A.S.; Ahsan, H.; Mamun, A.; Khanam, T.; Woods, J.; Salim, R.	Feasibility study of case-finding for breast cancer by community health workers in rural Bangladesh	2015	Asian Pac. J. Cancer Preven.	16	17	Bangladesh	A sub-district of the Khulna Division (eight villages)	Two primary aims: to determine the feasibility of using CHWs to identify serious breast problems and record patient data with and without cell-phone technology. Also, to determine if CHWs can increase access to medical care for patients with serious breast problems.
Duggan, C.; Dvaladze, A.L.; Tsu, V.; Jeronimo, J.; Constant, T.K.H.; Romanoff, A.; Scheel, J.R.; Patel, S.; Gralow, J.R.; Anderson, B.O.	Resource-stratified implementation of a community-based breast cancer management programme in Peru	2017	Lancet Oncol.	18	10	Peru	La Libertad	To implement a community-based program for breast health in order to improve breast healthcare delivery in Peru.
Earp, J.A.L.; Viadro, C.I.; Vincus, A.A.; Altpeter, M.; Flax, V.; Mayne, L.; Eng, E.	Lay Health Advisors: A strategy for getting the word out about breast cancer	1997	Health Educ. Behav.	24	4	United States	North Carolina	To use North Carolina Breast Cancer Screening Program’s (NC-BCSP) as a case study to describe the steps involved in developing, implementing, and evaluating a Lay Health Advisor (LHA) intervention aimed at transforming natural helpers into lay health advisors. The authors review five phases of implementation and how such an intervention bridges gaps between health care providers, agencies, and local communities.
Elder, J.P.; Haughton, J.; Perez, L.G.; Martínez, M.E.; De La Torre, C.L.; Slymen, D.J.; Arredondo, E.M.	Promoting cancer screening among churchgoing Latinas: Fe en Acción/Faith in Action	2017	Health Educ. Res.	32	2	United States	San Diego County, California	To evaluate self-reported cancer screening of a two-group randomized trial promoting breast, cervical, and colorectal cancer screening among churchgoing Latinas through *promotoras*. The article examines how screenings are affected by the community intervention Fe en acción/Faith in Action which was led by the *promotoras*.
English, K.C.; Fairbanks, J.; Finster, C.E.; Rafelito, A.; Luna, J.; Kennedy, M.	A socioecological approach to improving mammography rates in a tribal community	2008	Health Educ. Behav.	35	3	United States	New Mexico	To highlight the processes and intermediate outcomes of a pilot community-based intervention to increase mammography rates of women in an American Indian tribe in New Mexico.
Fernández, M.E.; Gonzales, A.; Tortolero-Luna, G.; Williams, J.; Saavedra-Embesi, M.; Chan, W.; Vernon, S.W.	Effectiveness of Cultivando la Salud: A breast and cervical cancer screening promotion program for low-income hispanic women	2009	Am. J. Public Health	99	5	United States	Anthony, NM; Eagle Pass, TX; Merced, California; Watsonville, California.	To increase breast and cervical cancer screening through an educational intervention for low-income Hispanic farmworker women who do not follow suggested guidelines for screening, and thereby reduce breast and cervical cancer incidence and mortality rates for Hispanic women.
Flax, V.L.; Earp, J.L.	Counseled women’s perspectives on their interactions with lay health advisors: A feasibility study	1999	Health Educ. Res.	14	1	United States	North Carolina (five Eastern rural counties)	To assess the feasibility of conducting semi-structured, in-depth interviews with black women of older age and to gather reports on women’s perceptions of the interactions with their Lay Health Advisors (LHAs).
Ford, S.; Meghea, C.; Estes, T.; Hamade, H.; Lockett, M.; Williams, K.P.	Assessing the fidelity of the Kin KeeperSM prevention intervention in African American, Latina and Arab women	2014	Health Educ. Res.	29	1	United States	Detroit, Michigan; Dearborn, Michigan	To examine fidelity and consistency of treatment delivery and see qualititatively the impact of the intervention.
Giarratano, G.; Bustamante-Forest, R.; Carter, C.	A multicultural and multilingual outreach program for cervical and breast cancer screening	2005	J. Obstet. Gynecol. Neonatal Nurs.	34	3	United States	New Orleans, Louisiana	To improve access to early detection screening of breast and cervical cancer amongst underserved, multicultural communities in New Orleans.
Gu, J.; Maxwell, A.E.; Ma, G.X.; Qian, X.; Tan, Y.; Hsieh, H.-C.; Tu, S.-P.; Wang, J.H.-Y.	Evaluating the training of Chinese-speaking Community Health Workers to implement a small-group intervention promoting mammography	2019	J. Cancer Educ.	34	4	United States	Washington, District of Columbia; Southern California; New York City, New York	To evaluate the effectiveness of the training curriculum and the fidelity of intervention implementions by Chinese-American CHWs on mammography rates among Chinese-American women in the community.
Gutnik, L.; Lee, C.; Msosa, V.; Moses, A.; Stanley, C.; Mzumara, S.; Liomba, N.G.; Gopal, S.	Clinical breast examination screening by trained laywomen in Malawi integrated with other health services	2016	J. Surg. Res.	204	1	Malawi	Lilongwe	To assess feasibility and performance of clinical breast exams by laywomen in urban health clinics in Malawi.
Han, H.-R.; Lee, H.; Kim, M.T.; Kim, K.B.	Tailored lay health worker intervention improves breast cancer screening outcomes in non-adherent Korean-American women	2009	Health Educ. Res.	24	2	United States	Los Angeles, California	To evaluate a model for training lay health workers to increase screening and education to detect breast cancer among in Korean-American (KA) women.
Han, H.-R.; Song, Y.; Kim, M.; Hedlin, H.K.; Kim, K.; Lee, H.B.; Roter, D.	Breast and cervical cancer screening literacy among Korean American women: A Community Health Worker-led intervention	2017	Am. J. Public Health	107	1	United States	Baltimore, Maryland and Washington, District of Columbia	To test a community health worker (CHW)-led health literacy intervention on mammogram and Pap test screening among Korean American women.
Huerta, E.E.; Weeks-Coulthurst, P.; Williams, C.; Swain, S.M.	Take care of your neighborhood	2018	Breast Cancer Res. Treat.	167	1	United States	Washington, District of Columbia	Assess the role of CHWs in countering the disparity of urban women who have insurance but do not seek care until have symptoms of advanced breast cancer.
Hunt, B.R.; Allgood, K.L.; Kanoon, J.M.; Benjamins, M.R.	Keys to the successful implementation of community-based outreach and navigation: Lessons from a breast health navigation program	2017	J. Cancer Educ.	32	1	United States	Chicago, Illinois	Assess effectiveness of CHW-led intervention in Chicago encouraging at-risk women to get mammograms and follow-up care as needed.
Kiger, H.	Outreach to multiethnic, multicultural, and multilingual women for breast cancer and cervical cancer education and screening: a model using professional and volunteer staffing.	2003	Fam. Community Health	26	4	United States	Santa Monica, California	To increase the number of women receiving breast cancer screening and to improve community education of breast health and awareness of screening services.
Kohler, R.E.; Miller, A.R.; Gutnik, L.; Lee, C.N.; Gopal, S.	Experiences and perceptions regarding clinical breast exam screening by trained laywomen in Malawi	2017	Cancer Causes Control	28	2	Malawi	Lilongwe	To explore participant perceptions of clinical breast exams as an intervention and to assess integrity of interventions in order to inform future program designs.
Larkey, L.K.; Herman, P.M.; Roe, D.J.; Garcia, F.; Lopez, A.M.; Gonzalez, J.; Perera, P.N.; Saboda, K.	A cancer screening intervention for underserved Latina women by lay educators	2012	J. Women’s Health	21	5	United States	Phoenix, Arizona	To test two methods of delivering a cancer screening and prevention curriculum taught by *promotoras*: individually or within a social support group.
Livaudais, J.C.; Coronado, G.D.; Espinoza, N.; Islas, I.; Ibarra, G.; Thompson, B.	Educating hispanic women about breast cancer prevention: Evaluation of a home-based promotora-led intervention	2010	J. Women’s Health	19	11	United States	Lower Yakima Valley, Washington State	To measure the impact of a home-based group educational intervention that addresses general cancer and breast cancer-specific awareness and screening.
Mayfield-Johnson, S.; Fastring, D.; Fortune, M.; White-Johnson, F.	Addressing breast cancer health disparities in the Mississippi Delta through an innovative partnership for education, detection, and screening	2016	J. Community Health	41	3	United States	Mississippi Delta	To increase breast cancer screening rates for African American women in the Mississippi Delta.
Nuño, T.; Martinez, M.E.; Harris, R.; Garcia, F.	A *Promotora*-administered group education intervention to promote breast and cervical cancer screening in a rural community along the U.S.-Mexico border: a randomized controlled trial	2010	Cancer Causes Control	22	3	United States	Yuma County, Arizona	To analyze the effectiveness of using lay community health workers (*promotoras*) to promote the utilization of breast and cervical cancer screening processes among post-menopausal Hispanic women.
Pace, L.E.; Dusengimana, J.-M.V.; Keating, N.L.; Hategekimana, V.; Rugema, V.; Bigirimana, J.B.; Costas-Chavarri, A.; Umwizera, A.; Park, P.H.; Shulman, L.N.; Mpunga, T.	Impact of breast cancer early detection training on Rwandan health workers’ knowledge and skills	2018	J. Glob. Oncol.	4	1	Rwanda	Burera District	To address the delays women experience between the onset of breast symptoms and first presentation at a health facility, as well as delays between symptom onset and receiving a breast cancer diagnosis.
Riehman, K.S.; Fisher-Borne, M.; Martinez, J.M.; Daven, M.; Thompson, L.; Fouad, M.N.; Partridge, E.E.	A Community Health Advisor program to reduce cancer screening disparities in the deep south and Appalachia: The American Cancer Society’s CHA collaborative	2017	Health Promot. Pract.	18	5	United States	Alabama, Arkansas, Kentucky, Louisiana, Mississippi, North Carolina, Tennessee, Virginia, and West Virginia	To reduce breast cancer screening disparities and increase community engagement, particularly in rural Appalachian and American-Indian populations. In addition, to train volunteers to deliver cancer prevention messages and accompany women to their screenings.
Scheel, J.R.; Molina, Y.; Briant, K.J.; Ibarra, G.; Lehman, C.D.; Thompson, B.	Latinas’ mammography intention following a home-based Promotores-led intervention	2015	J. Community Health	40	6	United States	Lower Yakima Valley, Washington State	To examine how knowledge and social engagement changed as a result of the home visits, and to determine if post-intervention knowledge and social engagement aligned with subsequent changes in mammography intention.
Schuster, A.L.R.; Frick, K.D.; Huh, B.-Y.; Kim, K.B.; Kim, M.; Han, M.-R.	Economic evaluation of a Community Health Worker-led health literacy intervention to promote cancer screening among Korean American women	2015	J. Health Care Poor Underserved	26	2	United States	Baltimore, Maryland; Washington, District of Columbia	To determine the cost-effectiveness of CHWs implementing interventions that aim to improve breast and cervical cancer screening rates.
Simon, M.A.; O’Brian, C.A.; Kanoon, J.M.; Venegas, A.; Ignoffo, S.; Picard, C.; Allgood, K.L.; Tom, L.; Margellos-Anast, H.	Leveraging an implementation science framework to adapt and scale a Patient Navigator Intervention to improve mammography screening outreach in a new community	2019	J. Cancer Educ.	N/A	N/A	United States	Chicago, Illinois	To evaluate the Helping Her Live (HHL) CHW-led model in the Southwest of Chicago (SW) and assess implementation of the program based on an already established program in the West side of Chicago (W)
Tum, S.J.; Maree, J.E.; Clarke, M.	Creating awareness and facilitating cervical and breast cancer screening uptake through the use of a Community Health Worker: A pilot intervention study	2013	Eur. J. Cancer Care	22	1	South Africa	Tshwane, South Africa	To increase rates of breast and cervical cancer screening, to increase community awareness of breast and cervical disease, and to evaluate the effectiveness of CHWs.
Welsh, A.L.; Sauaia, A.; Jacobellis, J.; Min, S.J.; Byers, T.	The effect of two church-based interventions on breast cancer screening rates among Medicaid-insured Latinas	2005	Prev. Chronic Dis.	2	4	United States	Denver, Colorado	To increase breast cancer screenings for Latinas in Colorado, using the church as a site of intervention due to its large impact in the social networks of the population.
Williams, K.P.; Mabiso, A.; Todem, D.; Hammad, A.; Hill-Ashford, Y.; Hamade, H.; Palamisono, G.; Robinson-Lockett, M.; Zambrana, R.E.	Differences in knowledge of breast cancer screening among African American, Arab American, and Latina women	2011	Prev. Chronic Dis.	8	1	United States	Detroit, Michigan; Dearborn, Michigan	To evaluate differences in breast cancer knowledge and socioeconomic status between the populations studied in association with multiple forms of breast cancer screening.
Williams, K.P.; Mullan, P.B.; Todem, D.	Moving from theory to practice: Implementing the Kin KeeperSM Cancer prevention model	2009	Health Educ. Res.	24	2	United States	Michigan	To evaluate if breast cancer education of individual women would lead to increased education among their families.
